# The Role of Tumor Microenvironment in Chemoresistance: To Survive, Keep Your Enemies Closer

**DOI:** 10.3390/ijms18071586

**Published:** 2017-07-21

**Authors:** Dimakatso Alice Senthebane, Arielle Rowe, Nicholas Ekow Thomford, Hendrina Shipanga, Daniella Munro, Mohammad A. M. Al Mazeedi, Hashim A. M. Almazyadi, Karlien Kallmeyer, Collet Dandara, Michael S. Pepper, M. Iqbal Parker, Kevin Dzobo

**Affiliations:** 1 Division of Medical Biochemistry and Institute of Infectious Disease and Molecular Medicine, Department of Integrative Biomedical Sciences, Faculty of Health Sciences, University of Cape Town, Cape Town 7925, South Africa; SNTDIM001@myuct.ac.za (D.A.S.); hmshipanga@gmail.com (H.S.); iqbal.parker@uct.ac.za (M.I.P.); 2 International Centre for Genetic Engineering and Biotechnology (ICGEB), Cape Town Component, Wernher and Beit Building (South), UCT Medical Campus, Anzio Road, Observatory, Cape Town 7925, South Africa; arielle.rowe@icgeb.org; 3 Pharmacogenetics Research Group, Division of Human Genetics, Department of Pathology and Institute of Infectious Disease and Molecular Medicine, Faculty of Health Sciences, University of Cape Town, Cape Town 7925, South Africa; thmnic023@myuct.ac.za (N.E.T.); MNRDAN002@myuct.ac.za (D.M.); collet.dandara@uct.ac.za (C.D.); 4 Batterjee Medical College, Prince Abdullah AlFiasal St, Obhur Al-Shamaliyah, Jeddah 23819, Saudi Arabia; almazeedi.mohammad@yahoo.com (M.A.M.A.M.); hashemmazeedi@gmail.com (H.A.M.A.); 5 Institute for Cellular and Molecular Medicine, Department of Immunology and South African Medical Research Council (SAMRC) Extramural Unit for Stem Cell Research and Therapy, Faculty of Health Sciences, University of Pretoria, Pretoria 0002, South Africa; karlienkallmeyer@gmail.com (K.K.); michael.pepper@up.ac.za (M.S.P.)

**Keywords:** chemoresistance, tumor microenvironment, tumor heterogeneity, mesenchymal stem cells, angiogenesis, extracellular matrix, clinical oncology

## Abstract

Chemoresistance is a leading cause of morbidity and mortality in cancer and it continues to be a challenge in cancer treatment. Chemoresistance is influenced by genetic and epigenetic alterations which affect drug uptake, metabolism and export of drugs at the cellular levels. While most research has focused on tumor cell autonomous mechanisms of chemoresistance, the tumor microenvironment has emerged as a key player in the development of chemoresistance and in malignant progression, thereby influencing the development of novel therapies in clinical oncology. It is not surprising that the study of the tumor microenvironment is now considered to be as important as the study of tumor cells. Recent advances in technological and analytical methods, especially ‘omics’ technologies, has made it possible to identify specific targets in tumor cells and within the tumor microenvironment to eradicate cancer. Tumors need constant support from previously ‘unsupportive’ microenvironments. Novel therapeutic strategies that inhibit such microenvironmental support to tumor cells would reduce chemoresistance and tumor relapse. Such strategies can target stromal cells, proteins released by stromal cells and non-cellular components such as the extracellular matrix (ECM) within the tumor microenvironment. Novel in vitro tumor biology models that recapitulate the in vivo tumor microenvironment such as multicellular tumor spheroids, biomimetic scaffolds and tumor organoids are being developed and are increasing our understanding of cancer cell-microenvironment interactions. This review offers an analysis of recent developments on the role of the tumor microenvironment in the development of chemoresistance and the strategies to overcome microenvironment-mediated chemoresistance. We propose a systematic analysis of the relationship between tumor cells and their respective tumor microenvironments and our data show that, to survive, cancer cells interact closely with tumor microenvironment components such as mesenchymal stem cells and the extracellular matrix.

## 1. Introduction

Cancer is a multifactorial disease and is one of the leading causes of death worldwide. It results from both genetic and epigenetic transformation of normal cells leading to abnormal proliferation. Cancer deaths outnumber the combined deaths from diseases such as HIV/AIDS, malaria and tuberculosis worldwide [1,2]. Despite the development of potent chemotherapeutics against many cancer types in recent years, durable or long lasting cure is still out of reach for many patients [3,4]. This is partly due to the development of drug/therapeutic resistance which stems from the remarkable adaptive behavior of cancer cells and is driven by both genetic and epigenetic factors. There are many distinct cancer types and these differ significantly in their genetic makeup, behavior and treatment responses [5]. Differences in cancer cells behavior stem from the dysregulation of a number of growth signals that are involved in the direct entry into and progression through the cell cycle. Due to the diverse nature of cancer, many treatment strategies have been developed and each takes advantage of a different aspect of the disease. However, most cancer drugs still target DNA replication and DNA repair mechanisms.

Cancer cells proliferate at a much higher rate than normal cells and invade nearby tissues or spread to distance organs. A number of oncogenes and tumor suppressor genes such as p53, c-Myc and transforming growth factor-β (TGF-β) are mutated in cancer cells and have been observed to play key roles in cancer cell proliferation, invasion and metastasis. Most of these oncogenes and tumor suppressor genes are considered as major contributors to drug resistance [6]. Resistance is usually accompanied by recurrence of the disease. Different cancer types respond to treatment in different ways and therefore some are better treated than others. The most common treatments for cancer are surgery, radiotherapy and chemotherapy. Surgery can successfully remove the cancerous tissue from the body and combined with chemotherapy and radiotherapy can be successful in treating any cancer [7]. Radiotherapy uses radiation to kill cancer cells. Chemotherapy remains the preferred method due in part to its effectiveness and low cost. Its lack of selectivity however hampers its success as normal cells are also killed in the process. Patients undergoing chemotherapy suffer many side-effects such as loss of hair, bleeding, nausea and death. Due to its genotoxic effects, chemotherapy induces changes in both normal and cancer cells creating further cancer cell heterogeneity and ultimately affecting the efficiency of chemotherapy [8].

A huge challenge in cancer treatment is the development of chemoresistance resulting in cancer cells that are more aggressive and able to metastasize [9]. Mechanisms that contribute to chemoresistance include tumor heterogeneity, drug inactivation, apoptosis evasion, enhanced deoxyribonucleic acid (DNA) repair, increased drug efflux, epithelial-to-mesenchymal transition and the involvement of the tumor microenvironment (TM) [8]. Though cancer cell chemoresistance is usually attributed to heterogeneity within the cancer cell population, mutations and epigenetic alterations influencing the metabolism and retention of drugs by cancer cells [10,11,12,13,14,15,16,17], additional causes could play important roles in the development of this phenomenon. Most important is the diversity within the tumor microenvironment (TM) in terms of the stromal cells present, the amount of oxygen available and the acidity of the environment [18,19,20,21,22,23,24]. Another important difference is the amount of the extracellular matrix (ECM) proteins around the cancer cells [25,26,27]. ECM proteins can create a barrier through which the drugs must pass in order to reach the cancer cells while their presence promote tumor metastasis [28,29,30,31,32,33,34]. As the tumor grows, it becomes difficult for chemotherapeutic agents to reach cancer cells near the center of the tumor. All these factors can have a huge influence on how cancer cells respond to drugs.

The genetic makeup of cancer cells and cellular processes occurring within cancer cells contribute immensely to the inability of most chemotherapeutic drugs used in clinical oncology to effectively clear these cells from the body [12,14,15,16,17,35,36,37]. Several mutations to key genes encoding important proteins responsible for xenobiotic metabolism, as well as import and export of drugs from cells such as the ABC transporters have been identified and shown to influence how tumor cells respond to several drugs [12,14,15,16,17,35,36,37]. However, with remarkable advancement in technology and analytical methods seen in the last decade, attention has shifted to the TM contribution towards the development of chemoresistance [38,39,40,41,42,43,44,45,46]. Chemotherapeutic drugs need to access all cancer cells in a solid tumor to be effective, thus components of the tumor microenvironment become important players in the response of these cells to drugs [33,47,48,49,50,51,52,53]. The TM is a dynamic entity and is characterized by cellular heterogeneous, the amounts of oxygen, nutrients and ECM proteins [41,54,55,56,57,58,59]. The heterogeneous nature of cancer and stromal cells within the TM is reflected in the ECM produced by these cells. The variability of the ECM within the TM also makes targeting the ECM difficulty and might explain why therapeutic targeting of the ECM has not had much success in several clinical trials. Both cancer cells and stromal cells do deposit the ECM in a tumor [60,61]. Novel strategies need to be developed to specifically target the ECM from different cells within the tumor. In addition, understanding the response of cancer cells to the ECM at different stages of tumor development would allow for the understanding of the contribution of each ECM protein during tumor progression. Determining the most effective time point when cancer cells respond to the ECM is also necessary in the intervention to stop cancer growth. In addition several studies have shown that matrix metalloproteinases play a huge role in inducing processes such as epithelial-mesenchymal transition with the end result being malignant transformation [60,61,62].

This review focusses on the contribution of the TM constituents in the development of chemotherapeutic resistance especially the role played by mesenchymal stem cells and the ECM. To overcome chemoresistance, it is imperative that the TM contribution be studied and specified as only then can we attain long lasting treatment in clinical oncology.

## 2. Cancer Cell Chemoresistance

The accumulation of genetic aberrations over time has been recognized as the main cause of cancer [63,64,65,66,67,68,69,70]. A combination of genetic mutations and epigenetic alterations results in tumor heterogeneity [67,71,72,73,74,75,76,77]. Tumor heterogeneity can contribute towards chemoresistance in many ways. Tumor heterogeneity is one of the recent addition to the list of drivers of chemoresistance [78,79]. Tumors are made up of cancer cells that differ in their phenotype and therefore chemotherapeutic responses. Differences in phenotypes may also arise due to cancer cell-microenvironment interactions besides the obvious genetic differences [78,79].

The implication of intratumor heterogeneity is that cancer cells within a tumor have different responses to the same chemotherapeutic drug. Variants of cancer cells that do not respond to a drug can result in relapse. Epigenetic modifications can take the form of DNA methylation and histone modification. Hypermethylation of the multi-drug resistance protein 1 (MDR1) gene promoter has been reported to cause downregulation of certain genes involved in apoptosis. Methylation of the O(6)-methylguanine DNA methyltransferase (MGMT) gene is known to cause silencing of several genes. A small fraction of undifferentiated cancer cells have anti-drug properties. These drug-resistant cancer cells are known to be present in circulation as well as in solid tumors. In addition, solid tumors have been shown to be a complex mixture of tumor cells, stromal cells and the ECM [80,81,82,83,84,85].

Chemotherapy destroys cancer cells mostly through induction of apoptosis by damaging DNA and inhibiting cell cycle progression [5,86,87]. Over time, cancer cells can acquire diverse strategies that decrease the efficacy of many therapeutic agents leading to chemoresistance [88]. Resistance to therapy occurs either as de novo or acquired. Acquired resistance occur when changes in the genetic makeup of cells over time result in therapy-resistant cells. De novo drug resistance can either be soluble-factor mediated drug resistance or cell-adhesion mediated drug resistance. Chemokines, growth factors and cytokines are known to induce the soluble factor mediated drug resistance. The interaction of cancer cells and stromal components such as fibroblasts and the ECM via surface receptors such as integrins induce cell-adhesion mediated drug resistance.

**Figure 1:**
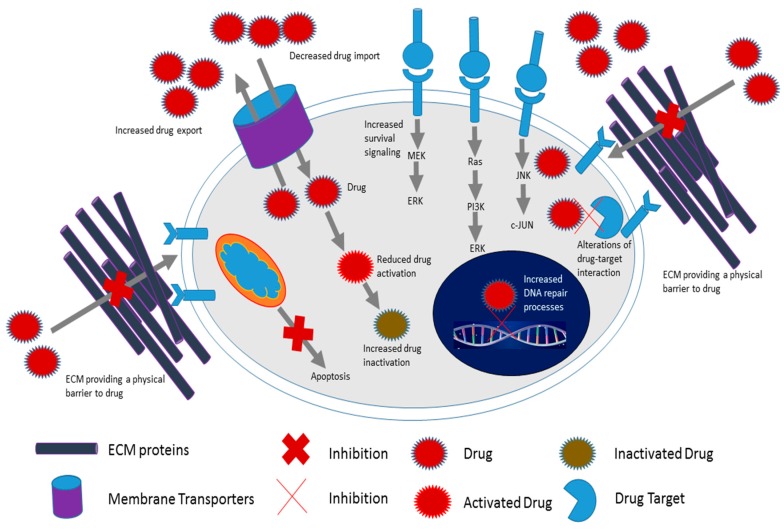
Schematic representation of processes that have been implicated in the development of chemoresistance. Some of these processes include enhanced survival signaling, enhanced drug inactivation, enhanced drug export, reduced drug uptake, inhibition of apoptosis, and increased production of extracellular matrix (ECM) proteins and inhibition of cytoskeleton organization (adapted from [92]).

The bi-directional communication between cancer and stromal cells is much more complex than initially perceived. Our data and that from others have shown that the cancer cell-stromal cell relationship is transient and ever changing [27,55,89,90]. Both tumor cells and stromal cells within the TM are exposed to different conditions over time including different concentrations of drugs. Eventually cancer and stromal cells develop a cooperative relationship that appear to benefit cancer cells. Through the release of soluble factors and the ECM, stromal cells determine the conditions within the TM. Stromal cells such as fibroblasts and mesenchymal stem cells have been the subject of many drug resistance studies to date. A summary of the various mechanisms known to be involved in cancer cell chemoresistance is shown in Figure 1. These mechanisms include enhanced survival signaling, enhanced drug inactivation, reduced drug uptake, enhanced DNA repair processes and inhibition of apoptosis [91].

## 3. Tumor Microenvironment

The dynamic nature of the TM during malignant progression underscores the need to study its role in disease progression. Importantly, the role of the cellular and non-cellular components in tumor initiation and progression needs to be investigated. Solid tumors are more than just a lump of cancer cells. Beside stromal cells, non-cellular components of the TM include the ECM and soluble growth factors [93,94,95,96,97,98]. The interaction between cells and their respective microenvironment is key for cellular growth and the maintenance of homeostasis. So it is for tumor growth. Though the gradual accumulation of genetic lesions creates the initial ‘spark’ necessary for disease initiation it is widely acknowledged that the TM play a critical role at every stage of malignant progression. Cancer cell-microenvironment interactions impacts on disease initiation, development and ultimately metastasis. Understanding the role of the TM in disease progression and chemotherapy is now considered central to cancer eradication. Initially thought to be only due to genetic lesions in cancer cells, the heterogeneous nature of tumors is now understood to be of microenvironmental origin as well. Both cellular and non-cellular components of the TM contribute towards the tumor heterogeneity observed in solid tumors. By contributing towards the tumor heterogeneity the TM components ultimately play a part in the development of chemoresistance. The crosstalk between tumor cells and their microenvironment makes this relationship very complex. However, the plasticity of the tumor stroma affords scientists an opportunity to devise therapeutic strategies that can allow most TM members to acquire anti-tumorigenic properties. It is also possible to convert pro-tumorigenic TM constituent members to become anti-tumorigenic.

**Figure 2:**
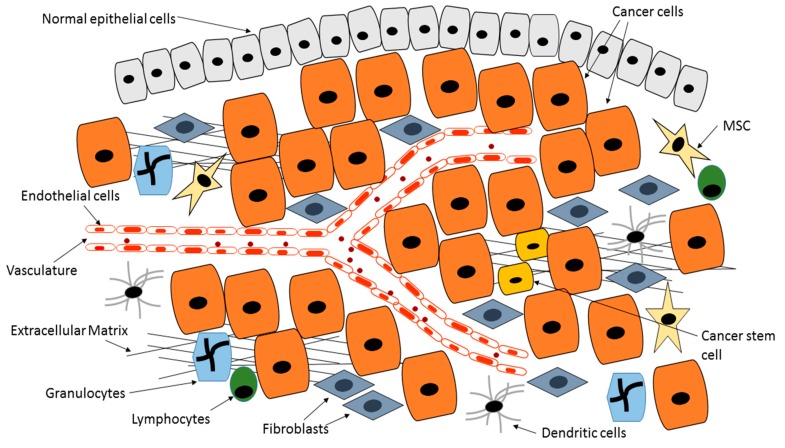
Tumor Microenvironment. The tumor microenvironment consists of several cells including cancer cells, mesenchymal stem cells (MSCs), endothelial cells, fibroblasts, cancer stem cells (CSC), bone marrow-derived cells (BMDC) as well as Extracellular matrix (ECM). All the cells in the tumor microenvironment (TM) contribute to tumor progression.

In normal tissues a homeostatic environment is maintained with most cells maintaining their differentiated states and well defined boundaries between tissue compartments. Tumor initiation and progression is associated with disruption of tissue architecture and organization [99,100,101]. An environment that was tumor inhibiting becomes permissive and supportive to tumor growth and metastasis [80,83,90,102,103,104]. The TM (Figure 2) is now identified as a leading factor that influences cancer cell proliferation, metastasis and anticancer drug efficacy [105,106,107]. Normal cellular processes and tissue homeostasis are reversed in tumors, as tumor cells bypass or override necessary homeostatic control measures. Cellular mechanisms of surrounding cells and the effect of non-cellular components is basically hijacked by cancer cells with the ultimate goal of ensuring cancer cells survival. Several anti-tumorigenic cells such as fibroblasts and macrophages are converted into tumor-promoting cells, releasing soluble factors such as growth factors and proteases needed by tumor cells to burrow through the ECM and support accelerated tumor cell growth [60,108]. Fibroblasts and macrophages are converted to cancer associated fibroblasts (CAFs) and tumor associated macrophages (TAMs) via the action of tumor-released factors such as TGF-β and platelet derived growth factor (PDGF). Both tumor-associated fibroblasts and macrophages are known to participate in this pro-tumorigenic process. Importantly CAFs are known to synthesize and deposit large quantities of thick ECM fibers, thus contributing to deregulated homeostasis. CAFs also contribute towards cancer cell invasion and metastasis through synthesis of metastasis-promoting ECM proteins such as fibronectin and periostin and the release of matrix metalloproteases. This allows tumor cells to lose their attachment to the ECM and acquire mesenchymal behavior. The origin of CAFs in solid tumor is controversial. The most straight forward suggestion is that they are of fibroblast origin. Through the action of tumor-derived factors normal fibroblasts are converted into ‘activated fibroblasts’ also termed CAFs with the function of bidding for tumor cell survival. Several studies have suggested that they are of endothelial origin. Yet other studies appear to show that mesenchymal stem cells can be converted to CAFs. Our studies support this suggestion. Tumor-released TGF-β appears to contribute to mesenchymal stem cells conversion to α-smooth muscle producing CAFs.

TAMs are known to locate to the leading edge of tumors where they release matrix metalloproteases needed to degrade the ECM. TAMs also contribute to the increased levels of growth factors such as EGF leading to tumor cell migration. The plasticity of macrophages allows them to act both as pro-tumorigenic and anti-tumorigenic depending on the surrounding environments and existing conditions. Through the release of pro-inflammatory cytokines, macrophages present antigens and play an anti-tumorigenic role in the TM. However, activated macrophages can be pro-tumorigenic through production of type II cytokines. Macrophages also help tumor cells intravasate into blood vessels. TM conditions such as hypoxia and acidity play significant roles in the activation of macrophages, with macrophages appearing to be attracted to regions of low oxygen tension. Localized selective pressures such as hypoxia and acidity select for stromal cells that ensure the survival of cancer cells. Several reports have also shown that the presence of growth factors and micro RNAs can drive activated macrophages back to normal leading to tumor regression. Thus resident macrophages can be targeted in the TM to have anti-tumorigenic properties. Due to the presence of several components within the TM, tumor cells are exposed to chemotherapeutic drugs in a gradient fashion. The ECM by forming thick fibers within the tumor present a physical barrier to diffusion of chemotherapeutic drugs [109,110,111,112,113,114].

The invasive nature of cancer cells cannot be exhibited without the interplay between tumor cells and their microenvironment. With an increase in our knowledge of molecular targets and medicine, clinical therapeutic targets have increased to include components of the TM [28,54,89]. The crosstalk between stromal and tumor cells involves growth factors, chemokines and cytokines as well as ECM and can affect the sensitivity of anticancer drugs and pathways involved in apoptosis [115,116]. Tumors depend on the formation of new blood vessels, through a process called angiogenesis, in order to increase in size as tumor cells need a constant supply of oxygen and nutrients [117,118,119]. Due to tissue disorganization associated with tumors, they tend to have lower blood flow and therefore less drugs reaches the tumor cells than is administered.

For chemotherapeutic drugs to reach tumor cells in vivo, they have to travel through the blood vessels [120,121,122,123]. Blood flow through a solid tumor is varied and disorganized [124,125]. Most blood vessels in tumors are dilated and “leaky” compared to those in normal tissues. Thus the vasculature also influences the response of tumor cells to drugs. The compactness of a solid tumor increases blood flow resistance and this causes gradients of nutrients and oxygen, meaning that there are different proliferation rates in different regions of the tumor [126,127,128,129,130,131]. Nutrient deprivation is also a result of blood flow resistance. Most chemotherapeutic drugs are designed against highly proliferating cells and not quiescent cells [132,133,134,135]. Thus having tumor cells proliferating at different rates has a major impact on the effectiveness of chemotherapy. Due to impaired blood flow the clearance of breakdown products within the tumor can lead to a toxic microenvironment [136,137,138].

The compactness of a solid tumor and the reduced blood flow leads to either temporary or chronic hypoxia [139,140,141,142,143,144,145]. Cells in hypoxic conditions tends to divide slowly, making them unresponsive to chemotherapeutic reagents. Hypoxia can result in the activation of genes associated with angiogenesis and cell survival [146,147,148]. Many chemotherapeutic drugs cause DNA damage through generating free radicals [149,150,151]. Without oxygen however, the cytotoxicity of several chemotherapeutic drugs whose activity depends on generating free radicals is reduced. In addition, it has been shown that cancer stem cells (CSCs) tend to be located at the center of a solid tumor [152,153,154,155,156,157]. These CSCs are able to withstand lower oxygen levels than the general population of cancer cells [18,80]. Thus the inability of drugs to reach the center of the solid tumor can result in recurrence of the tumor even after an apparent successful treatment. It has also been observed that hypoxia can lead to increased expression of P glycoprotein, which is involved in drug inactivation, resulting in drug resistance [158]. Hypoxia inducible factor (HIF)-1 is stimulated under low oxygen conditions and this transcription factor controls many genes involved in survival mechanisms such as angiogenesis and apoptosis. Several pro-drugs have been developed to be activated under complete or partial hypoxic conditions. For example, Tirapazamine (TPZ) is activated over a range of oxygen levels. Due to the varying amounts of oxygen in solid tumors, TPZ activation over time. Thus at some point tumor cells are exposed to sub-lethal levels of TPZ with the consequent development of chemoresistance. The pH in the TM can affect the cytotoxicity of anticancer drugs. An acidic microenvironment can inhibit the activation of many chemotherapeutic drugs [159,160]. Changes in pH inside and outside of cancer cells can have a lasting effect on chemotherapeutic drugs. The pressure gradient that exists within the microenvironment also influences the distribution of many anticancer drugs. The ability of cancer cells to manipulate their microenvironment enables them to acquire important hallmark properties that are necessary for tumor growth and development.

### 3.1. Cancer-Associated Fibroblasts (CAFs)

Cancer-associated fibroblasts (CAFs) are activated fibroblasts found in association with cancer cells. CAFs are the most abundant cells within the TM and are involved in tumor initiation, by activating signals involve in growth and differentiation, and evade cancer therapy [161,162]. CAFs secrete growth factors, such as hepatocyte growth factor (HGF), epidermal growth factor (EGF), and cytokines such as stromal cell-derived factor 1 (SDF-1) and IL-6. Wang et al. showed that secretion of HGF by CAFs induced resistance to EGF-tyrosine kinase inhibitors in lung cancer cells [163]. Secretion of chemokines and cytokine by CAFs can lead to immune cells infiltration which contribute to angiogenesis and metastasis [164]. CAFs are known to stimulate the growth of new blood vessels through the release of growth factors such as vascular endothelial growth factor. Enhanced invasion of pancreatic cancer cells was observed in the presence of fibroblast-derived SDF-1 and IL-8 was also found to induce angiogenesis in vitro [165]. CAFs can regulate ECM composition via expression of matrix metalloproteinases (MMPs), which allows cancer cell adhesion and migration as well as inhibition of apoptosis by activating PI3K-Akt/PKB as seen in breast cancer models [158]. The presence of CAFs or transformed fibroblasts is known to activate migratory behavior in cancer cells through upregulation of integrin expression and cell survival signaling pathways such as the MEK-ERK and the PI3K-Akt pathways. In prostate cancer, increased secretion of MMP-2 and MMP-9 by CAFs was associated with the induction of epithelial-mesenchymal transition (EMT), known to be involve in cancer cell invasion and metastasis [166]. CAFs also secrete IL-6, which promotes cancer metastasis and chemoresistance through induction of EMT [167]. A study by Conze and colleagues showed that IL-6 is overexpressed in multidrug resistant breast cancer [168]. In vitro and in vivo studies have shown that CAFs derived from breast cancer induced tamoxifen resistance through decreasing estrogen receptor-α (ER-α) levels and IL-6 secretion [169].

### 3.2. Mesenchymal Stromal/Stem Cells (MSCs)

MSCs have received a lot of attention in cancer biology partly because of their primitive nature and their ability to generate several other cells types. Through the action of tumor cell-derived factors, MSCs are recruited to the tumor site where they produce factors needed by cancer cells. MSCs are found in many adult tissues including bone marrow and adipose tissues [170]. MSCs can self-renew and differentiate into specialized tissue-specific cell types such as adipocytes, chondrocytes, fibroblasts and osteoblast [167,170,171,172]. MSCs are also found in the TM and are known to play an important role in tumor progression and chemoresistance [172]. MSCs promote tumor growth either by the secretion of growth factors, or by differentiating into tumor associated fibroblasts (TAFs) [55,170,173,174]. The origin of TAFs or CAFs in the TM is still debatable. TAFs are a heterogeneous cell population and are commonly identified by α-smooth muscle actin (α-SMA) and vimentin expression which is indicative of an ‘activated’ myofibroblast-like phenotype [175,176]. One source of TAFs that has been touted is MSCs present in the tumor stroma [175,176]. We present data from an extension of our previous publication [90], showing that long term co-culture of esophageal WHCO1 and breast cancer MDA MB 231 cells with human MSCs trigger hMSCs differentiation into ‘tumor associated fibroblasts’ via the TGF-β/Smad signaling pathway.

**Figure 3:**
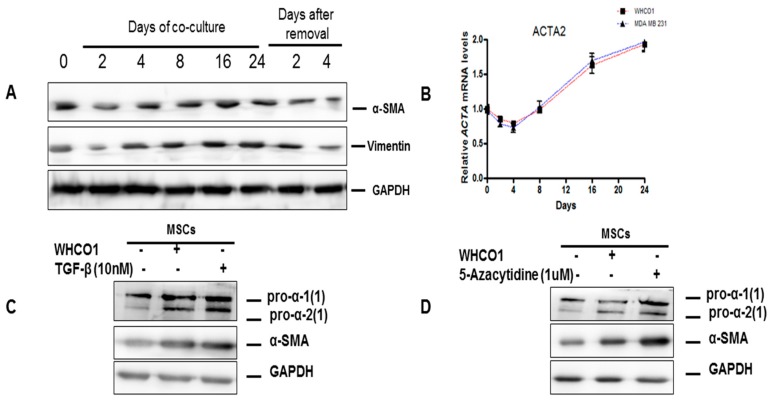

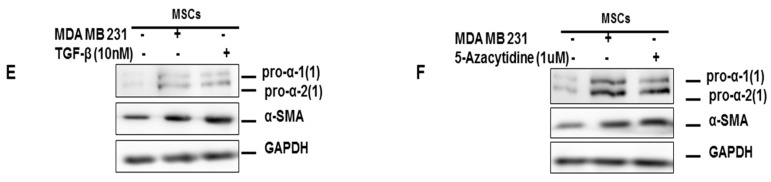
Cancer cells trigger MSCs differentiation into tumor associated fibroblasts via the transforming growth factor-β (TGF-β) /Smad signaling pathway. For the co-culture experiments cells were co-cultured in 6-transwell plates (size of pore: 0.4 μm, Polycarbonate membrane, Costar, Corning, Cambridge, MA, USA). Mesenchymal stem cells (5 × 10^5^ cells) were cultured in the upper insert and cancer cells (WHCO1 and MDA MB 231) (5 × 10^5^ cells) were cultured in the lower compartment. Empty inserts were used for the control group (no cells) and a mixture of MSCs medium and cancer cell medium (1:1) was used. Medium was changed every 3 days for longer incubation periods and fresh TGF-β and reagents were added. TGF-β and all reagents were added to the media to the final concentrations as shown. At specific time points or at the end of the experiment, cells (cancer cells and MSCs) were harvested and used in various analyses. (**A**) Western blot analysis of lysates from MSCs co-cultured with WHCO1 cells for 24 days showing α-smooth muscle actin (α-SMA) and vimentin protein levels. Glyceraldehyde 3-phosphate dehydrogenase (GAPDH) was used as a loading control. (**B**) Real time quantitative reverse transcription polymerase chain reaction (RT-qPCR) analysis was performed to assess the expression of Actin, alpha2, smooth muscle, aorta (ACTA2) (α-SMA gene) in MSCs co-cultured with WHCO1 and MDA MB 231 cancer cells over a 24 day period; (**C**,**D**) western blot analysis of lysates from MSCs co-cultured with WHCO1 cells for 16 days or after the addition of 10 nM TGF-β (**C**) or 1 μM 5-azacytidine (**D**) for 48 h showing the expression of type I collagen and α-SMA; (**E**,**F**) western blot analysis of lysates from MSCs co-cultured with MDA MB 231 cells for 16 days or after the addition of 10 nM TGF-β (**E**) or the addition of 1 μM 5-azacytidine (**F**) for 48 h showing the expression of type I collagen and α-SMA.

**Figure 4:**
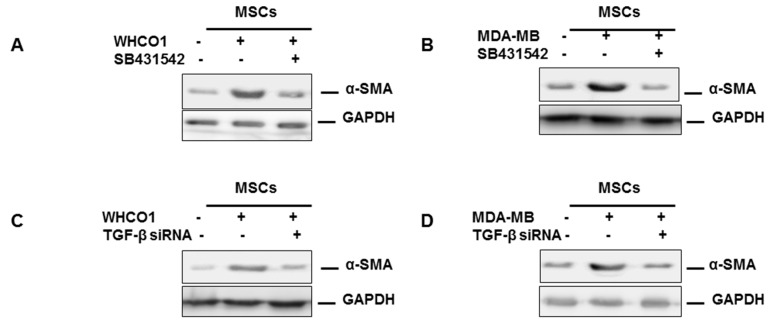

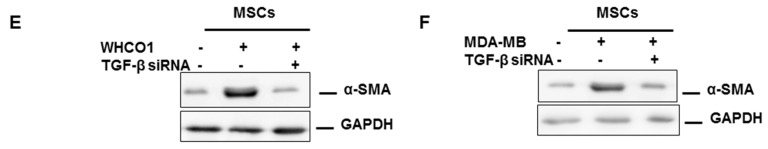
WHCO1, MDA MB 231 cells and MSCs secrete TGF-β. Mesenchymal stem cells (5 × 10^5^ cells) were cultured in the upper insert and cancer cells (WHCO1 and MDA MB 231) (5 × 10^5^ cells) were cultured in the lower compartment as described in Figure 3. At specific time points or at the end of the experiment, cells (cancer cells and MSCs) were harvested and used in various analyses. (**A**,**B**) TGF-β inhibitor SB431542 was added to the co-culture media to a final concentration of 10 µM. Co-culture was continued for 16 days after which α-SMA protein levels was determined by western blot analysis. Glyceraldehyde 3-phosphate dehydrogenase (GAPDH) was used as a loading control. (**C**,**D**) MSCs were treated with TGF-β siRNA to a final concentration of 100 nM and co-culture was continued for 16 days. To maintain knockdown of TGF-β, subsequent transfections were done every other three days till the end of the experiment. Western blot analysis was performed to evaluate the α-SMA protein levels in MSCs lysates; (**E**,**F**) WHCO1 and MDA MB 231 cells were treated with TGF-β siRNA to a final concentration of 100 nM and co-culture was continued for 16 days. To maintain knockdown of TGF-β, subsequent transfections were done every other three days till the end of the experiment. Western blot analysis was performed to evaluate the α-SMA protein levels in MSCs lysates.

In our study, we evaluated the effect of esophageal WHCO1 and breast MDA MB 231 cancer cells on Wharton’s Jelly-derived mesenchymal stromal/stem cells (WJ-MSCs) gene expression over 24 days of co-culture. The expression of α-SMA, the most reliable marker of tumor associated fibroblasts (TAFs) and vimentin showed a clear and gradual increase in WJ-MSCs up to a maximum at day 16 in our co-culture system (Figure 3A,B). TGF-β is one of the growth factors released by cancer cells in order to evade immune detection in vivo and can increase expression of proteins such as α-SMA and vimentin. Treatment of WJ-MSCs with 1 µM 5-azacytidine resulted in their differentiation into myofibroblastic lineages expressing increased levels of α-SMA and type I collagen. Addition of exogenous TGF-β (10 µM) and treatment of naïve MSCs with 5-azacytidine (1 µM) up to 48 h resulted in increased levels of α-SMA and type I collagen similar to MSCs co-cultured for 16 days (Figure 3C–F). Our observations show that over time MSCs exposed to esophageal and breast cancer cells differentiate and express markers of the myofibroblastic lineage. Many studies have shown that ACTA2 (α-SMA) gene transcription is regulated through the interactions of several signaling pathways. To substantiate these results, the TGF-β inhibitor SB 431542 (10 nM) was added to the co-culture media. Addition of SB 431542 decreased the α-SMA protein levels in MSCs exposed to WHCO1 and MDA MB 231 cells (Figure 4A,B). As an orthogonal approach, suppression of TGF-β expression in co-cultured MSCs through the use of TGF-β siRNA resulted in decreased α-SMA protein levels (Figure 4C,D). In addition, TGF-β knockdown in both WHCO1 and MDA MB 231 cells during co-culture decreased α-SMA protein levels in MSCs (Figure 4E,F). We also observed that Smad2 increased in WJ-MSCs cocultured with WHCO1 and MDA MB 231 cells (data not shown). These results demonstrate that the TGF-β/Smad signaling pathway is involved in the differentiation of MSCs into TAFs and that TGF-β probably is probably produced by both MSCs and cancer cells.

**Figure 5:**
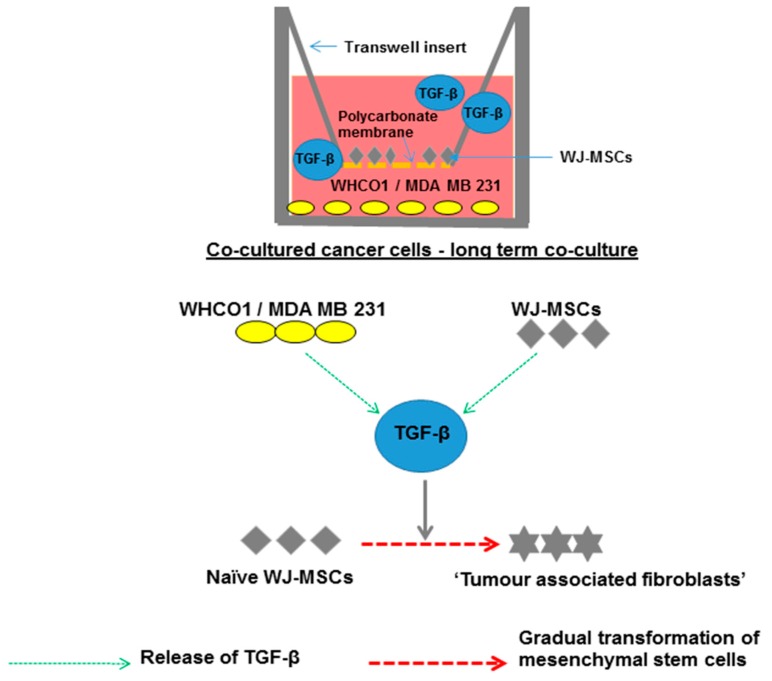
Our co-culture experiments have shown that TGF-β plays an important role in the interaction between cancer cells and MSCs. Our data show that in the long term, WHCO1 and MDA MB 231 cancer cell exposed-Wharton’s Jelly derived-MSCs differentiate into tumor associated fibroblasts (TAFs) through a TGF-β/Smad-mediated process.

**Figure 6:**
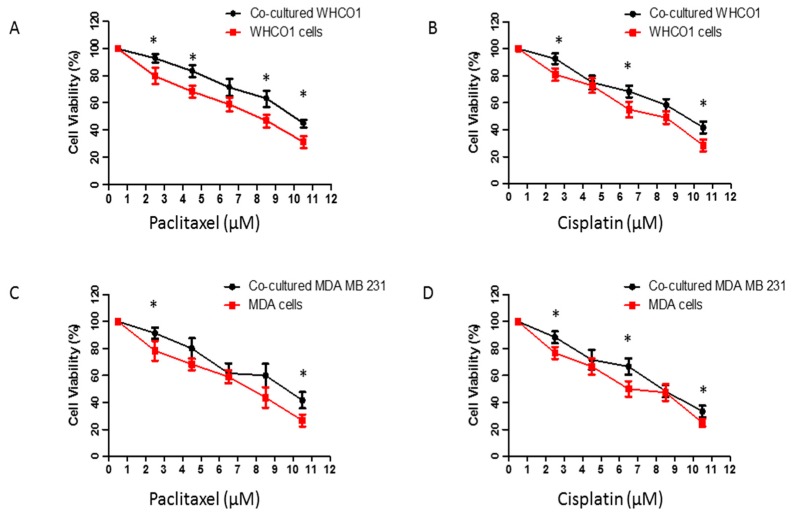
Co-cultured cancer cells survive treatment with cisplatin and paclitaxel better than WHCO1 and MDA MB 231 cells alone. WHCO1 and MDA MB 231 cancer cells (5 × 10^5^) were cultured alone or co-cultured with WJ-MSCs for 16 days as described in Figure 3. Empty inserts were used for the control group (no MSCs) and a mixture of MSCs medium and cancer cell medium (1:1) was used. Medium was changed every 3 days for longer incubation periods. At the end of the incubation, the same number of WHCO1 and MDA MB 231 cancer cells were treated with increasing concentrations of paclitaxel and cisplatin for 48 h as shown above. After 48 h, cells were counted with a Countess Cell counter using the Trypan Blue exclusion method. Cells were expressed as a percentage of cells treated with 0.1% DMSO (control). Experiments were repeated three times. * *p* < 0.05.

Thus it is possible that cancer cells can attract MSCs to the tumor site and the MSCs can become part of the TM as TAFs. However other cells can also be a source of TAFs. TAFs are known as accomplices in increased tumor growth, metastasis and chemoresistance [177,178]. MSCs can also promote drug resistance both by secreting protective cytokines, and by preventing cancer cell apoptosis [177]. Our data show that MSCs can be transformed to CAFs by cancer cells through release of growth factors such as TGF-β (Figure 5). Importantly, both WHCO1 and MDA MB 231 cells co-cultured with “cancer cell activated” WJ-MSCs survive treatment with paclitaxel and cisplatin better than WHCO1 and MDA MB 231 cancer cells alone (Figure 6).

Both WHCO1 and MDA MB 231 cancer cells co-cultured with the above WJ-MSCs for 16 days survived treatment with cisplatin and paclitaxel better than WHCO1 and MBA MB 231 cell alone (Figure 6). It is evident that the presence of WJ-MSCs, possibly through the release of protein factors, protected the cancer cells from the effect of the drugs used.

### 3.3. The Role of the Extracellular Matrix in Chemotherapeutic Resistance

The ECM is the crucial non-cellular component of the TM and consists of mainly glycoproteins, proteins and proteoglycans [179]. The ECM plays key roles in tissue maintenance and function. The ECM regulates cellular behavior directly and indirectly [179]. Due to the crucial roles the ECM plays in vivo, a number of mechanisms are involved in the regulation of ECM production, degradation and remodeling [180]. Perturbation of these mechanisms can promote pathological conditions such as fibrosis and cancer [179,181]. The physical properties of the ECM determines its role as a scaffolding to maintain tissue structure and function [179]. It also controls the behavior of cells through proliferation, differentiation and signaling pathways [182,183]. The signaling abilities of the ECM’s biochemical properties permits interactions between cells and their environment [179]. The composition and structure of the ECM is precisely tuned according to the needs of the surrounding cells. This is achieved through the release of soluble factors such as growth factors and chemokines. Besides serving as a physical scaffold onto which cells are anchored, the ECM provides signals necessary for cellular growth, migration and differentiation. Both physical and chemical properties of the ECM can influence cellular behaviors and these properties can be altered in cancer. ECM remodeling involves many enzymes, including matrix degrading enzymes including MMPs, lysyl oxidase (LOX), tissue inhibitors of metalloproteinases (TIMPs) and cathepsins [60]. Thus the composition of the ECM in cancer is a very important factor in deciding the efficacy of many drugs. Most cancer cell behavior is affected by the surrounding ECM. Effective cancer treatment requires knowledge of the cancer-ECM interactions in addition to the interactions with other TM components.

Due to its plasticity, the ECM has been ascribed both pro-tumorigenic and anti-tumorigenic properties. Initially thought to be a passive bystander, the ECM is emerging as a key player in malignant initiation, progression and chemoresistance. It is likely that the ECM inhibits early tumor growth and at later stages becomes pro-tumorigenic. Several studies have shown that the ECM present in the TM influence disease progression and is a major indicator of clinical prognosis. High levels of protease inhibitors within the ECM is associated with a good clinical outcome whilst high levels of surface receptors such as integrins and MMPs are associated with a poor outcome and relapse of disease. The ECM and its associated proteins, now referred to as the ‘matrisome’, is synthesized by different types of cells within the TM. The manipulation of the ECM and its ligands offers an attractive therapeutic avenue to eradicate cancer. Many studies have shown that matrix stiffness can influence cellular adhesion to surfaces, migration, differentiation and even proliferation [27,89,184,185,186]. Cells migrating to other regions have been shown to be softer and more pliable than benign cells. In general the tumor surrounding-ECM has been found to be stiffer than the ECM surrounding healthy tissues [23,51,186,187,188,189,190,191]. The stiffening observed in cancer is thought to be linked to fibrosis and deposition of collagen as shown in breast cancer [139,192,193,194,195]. Studies have showed that a stiff microenvironment induces tumor progression and malignancy through integrin signaling as a result of ECM tumor-associated remodeling [106,107,179,196]. Several studies have reported that tumor metastasis is promoted by ECM stiffening through the action of lysyl oxidase and the increased deposition of collagens and fibronectin. Stiffened ECM downregulates the expression of genes associated with cell cycle inhibition. MicroRNAs are reportedly induced by matrix stiffening and these microRNAs downregulates the expression of PTEN, a tumor suppressor protein. This has the effect of increasing PI3K-Akt activity, a survival pathway implicated in tumor growth and metastasis. Inhibition of lysyl oxidase (LOX) softens the ECM [197]. ECM abnormality is known to result in cancer cell growth, survival, migration and anticancer drug resistance [179]. However, the ECM is composed of many constituents and as such it is difficult to pinpoint the role of each component on tumor progression. It is likewise difficult to recapitulate the in vivo situation in an in vitro setting in order to study the effect of each individual TM component on tumor progression and chemoresistance.

Several ECM proteins have been associated with resistance to chemotherapy. Fibronectin has been associated with increased migration of several cancer cells [198,199,200,201]. Changes in ECM elasticity and stiffness are some of the factors known to affect drug delivery to cancer cells. ECM stiffness has been associated with tumor initiation in many cancer types. Dysregulation of ECM remodeling can result in the evasion of apoptosis by mutant cells, enlargement of CSC pool and disruption of tissue polarity [179]. Like normal cells, tumor cells need nutrients, oxygen and waste exchange [179]. These needs are met by angiogenesis, which results in increase in tumor size, and by lymphangiogenesis, the growth of lymphatic blood vessels [179]. Diffusion and pressure are associated with drug delivery in the interstitial spaces. ECM remodeling promotes drug resistance in the form of physical barrier that dissolve or delay drug delivery [202]. Interaction of the ECM with other cells has been considered to be involved in the promotion of chemoresistance through the activation of survival proteins [203]. These survival pathways include PI3K/AKT, p53 and MAPK which have been demonstrated to be activated upon the binding to ECM. Cancer should no longer be viewed as a disease of mis-regulated or mutated genomes. That tumors are like organs is illustrated more by their dependent on angiogenesis. The tumor’s need for nutrients and oxygen, supplied via the bloodstream, makes angiogenesis a necessity for tumor growth. Several factors are released by stromal cells within the TM to initiate and to allow tumor vascularization to occur.

#### 3.3.1. Collagen

Collagen is the main ECM protein synthesized in several tissues. Collagen is known to promote cancer cell clustering and invasiveness. Collagen is an ECM protein for scaffolding and provides tissue strength and support. The structural organization and level of collagens within tissues can indirectly influence drug efficacy. Type I and IV collagen can promote drug resistance through the interaction with integrins on cancer cells [204,205]. An environment rich in collagen is known to activate several signaling pathways such as MEK-ERK and the Wnt/B-catenin pathways. Increased expression of ECM proteins such as collagen by cancer cells further limits the diffusion of chemotherapeutic drugs into cancer tissues [206,207,208]. Drug delivery is significantly limited by tortuous and dense tumor ECM [207,208]. The expression of collagen play a crucial role in both drug resistance at the cellular level and tissue mediated drug resistance [206]. Interaction of ECM proteins including collagens with cancer cells can alter the cancer cell response to the presence of chemotherapeutic reagents [206,209]. Several studies have shown that high levels of expression of collagen genes was associated with drug resistance in ovarian and breast cancer cell lines [206,210]. The time needed for drugs to penetrate through collagen fibers before reaching cancer cells is lengthened, which can result in drug resistance [211].

ECM that contains large amount of collagen enhances tumor progression and invasiveness [204,210,212]. Pancreatic ductal adenocarcinoma (PDA) is one of the most aggressive human malignancies and a leading cause of cancer mortality. A unique molecular hallmark associated with PDA is the presence of dense collagen-rich fibrosis [213]. Increased expression of type I collagen has been associated with increased risk of metastasis in several cancer types [213,214,215]. Pancreatic cancer cells cultured in 3D collagen showed decreased sensitivity to gemcitabine therapy and increased proliferation despite drug treatment [213,216]. Collagen type XI α1 (COL11A1) is a member of collagen family, which is the important component of the interstitial ECM. Overexpression of COL11A1 is associated with progression of several cancers and poor survival [177,178,217]. COL11A1 expression has been demonstrated to be high in cisplatin-resistance ovarian cancer cells [218,219]. In addition, COL11A1 promotes ovarian cancer cell chemoresistance through the activation of signaling pathways such Akt and PDK1 pathways [219]. The deposition of collagens, expression of LOX and increased ECM stiffness in breast cancer resulted in increased adhesion and PIK3 activity [197]. These findings suggest that the TM induces chemo-protection and increases cancer cell survival through remodeling of components in the ECM.

#### 3.3.2. Laminin

Laminin constitutes a major family of the ECM proteins in the basal lamina and is known to affect cellular processes such as differentiation, adhesion and migration [220,221]. This family of ECM proteins plays a key role in the invasive behavior of several cancer cells. Laminin-332 (LN-332) is a heterotrimer made of β3, α3 and γ2 chains that has been shown to be key in cell adhesion and cancer metastasis [220,221,222,223]. Laminin-332 is involved in maintaining the self-renewal abilities of CSCs and has been implicated in resistance to sorafenib and doxorubicin [223]. Laminin β3 chain expression is associated with poor outcome in colorectal cancer and is related to chemoresistance to 5-FU-based chemotherapy regimens [222]. Another family member, Laminin β1 (LAMB1), is increased in paclitaxel-resistance cell lines [220].

LN-332 can bind the integrin α3β1 receptor which is reported to be enhanced in gefitinib resistance in hepatocellular carcinomas (HCCs) [224]. LN-integrin interactions increase cell survival and chemoresistance through the activation of mTOR [223,225]. It was demonstrated that LN-332 does not only protect hepatic cancer cells against therapeutic drugs but it promotes cell proliferation upon sorafenib exposure [223]. LN-332 and its γ2-chain play a key role in CSC self-renewal and differentiation and in maintaining and supporting quiescence as part of the human hepatic cancer stem cell niche [223]. LN protects pancreatic cells from gemcitabine induced apoptosis and cytotoxicity [226]. The protection of pancreatic cells by LN is a result of the activation of focal adhesion kinase (FAK), itself a result gemcitabine resistance induced apoptosis [226].

#### 3.3.3. Fibronectin

Fibronectin (FN) plays a crucial role in growth, differentiation, adhesion and migration [227]. Fibronectins are glycoproteins that attach cells to collagen fibers in the ECM, facilitating movement of cells through the ECM [227]. Fibronectin binds to cell surface integrins and collagen resulting in reorganization of the cell’s cytoskeleton allowing movement of cells. Fibronectin has been found to be key in wound healing and in cancer initiation and progression [200]. Increased tumorigenicity and resistance to apoptosis-inducing therapeutic drugs in lung cancer is achieved when lung carcinoma cells adhere to FN [228]. Overexpression of FN at the invasion front and in tumor stroma is observed in head and neck squamous cell carcinomas (HNSCCs) [229]. Increased expression of FN in HSCC is associated with decreased survival of patients [200]. FN induced migration of carcinoma collectives through αvβ6 and α9β1 integrins [200]. Small-cell lung cancer cells (SCLC) that adhered to laminin, collagen and fibronectin were found to be protected from apoptosis induced by chemotherapeutic drugs compared to those that were grown on plastic [228]. FN facilitated non-small cell lung carcinoma cell (NSCLC) growth and reduced apoptosis through induction of cyclooxygenase-2 (COX-2) and activation of integrin α5β1 [230]. These effects were correlated with activation of many kinase signaling pathways such as MEK-ERK and Rho kinase signaling pathways [231]. FN adhesion led to protection of tumor cells against docetaxel-induced apoptosis [223,231,232].

#### 3.3.4. Periostin

Periostin, a secretory protein also known as osteoblast-specific factor 2, is expressed as an extracellular matrix protein [233,234]. It is a cell adhesion protein that plays important roles in tooth and bone tissue homeostasis and development. It has also been found to be key in cardiac development and healing [220,233,234]. Overexpression of periostin has been implicated in many types of cancer such as gastric, colon, esophageal, ovarian, thyroid, lung, breast and head and neck carcinomas [233,234]. It regulates cell-matrix interactions through binding to fibronectin, type I/V collagen and tenascin C [234]. Periostin is a ligand for integrins such as αvβ3, αvβ5 and α6β4 [235]. It interacts with several signaling pathways such as Notch 1 and B-catenin signaling. It has been demonstrated that periostin in normal esophagus is significantly lower than in esophageal squamous cell carcinoma (ESCC) [234,235]. Periostin induces the PI3K-Akt signaling pathway by binding as a ligand to αvβ3 and αvβ5 integrins in esophageal cancer [176]. Periostin also increases cancer cell proliferation and EMT in nicotine-induced gastric cancer [176]. Periostin is overexpressed in gastric cancer cells that are resistance to cisplatin and 5-fluorouracil (5-FU) [234]. It was shown that periostin levels correlated with tumor angiogenesis and tumor recurrence [236]. In epithelial ovarian carcinoma, periostin induced Akt phosphorylation to increase resistance to paclitaxel [237]. Periostin not only serves as a prognostic factor for clinical outcome but also plays a role in resistance in several tumor cell types [238].

## 4. Strategies to Overcome Chemoresistance

To date most remedies for cancer have tended to focus directly on intrinsic characteristics of cancer cells. This is despite the fact that a tumor is a heterogeneous mixture of cancer cells, stromal cells and the extracellular matrix. Indeed, heterogeneity occurs at every level in cancer cells. Targeting stable stromal cells, with less or no genetic mutations, therefore is appealing. Stromal cells, due to their stable genetic makeup, are less likely to develop resistance to therapeutic agents. Perturbing or removing all the supporting cells and non-cellular components in the TM should ultimately lead to tumor regression or tumor cell reversion. Most stromal components can be engineered to be anti-tumorigenic due to their pliable behaviors. Given the heterogeneity evident in all cancers, it is imperative to study the TM as a possible avenue for cancer treatment. Indeed, several reports on combination therapies against both cancer and stromal cells appear to show promising outcomes in animal studies and in early phases of clinical trials. Several important aspects of TM-directed therapies need to be researched further. For example, the use of MSCs in cancer treatment needs to be studied further as several studies have shown that over time MSCs can be converted to ‘cancer associated fibroblasts’. Thus benefits derived from MSCs might be negated in the long run if MSCs are converted to CAFs. We advocate for the inclusion of TM components in in vitro experimental systems in order to delineate the role of TM components on cancer cell growth and metastasis. Novel animal models that are able to initiate tumors within native tissues will advance our understanding of the involvement of the TM in malignant initiation and development.

The efficacy of chemotherapeutic drugs may be impaired in several ways including limited delivery of drugs, cell death inhibition, drug inactivation, drug target alteration, EMT, the involvement of the TM or any combination of these factors. Therefore, combination therapy appear to be a reasonable solution to prevent drug resistance in many cancer types. Several novel ways have been suggested to overcome drugs resistance due to microenvironmental factors. Before treatment, administration of antiangiogenic therapy can help to remove extra capillaries and abnormal blood vessels leading to a reduction in the pressure of the interstitial fluid [239,240,241,242]. Other suggestions include damaging already existing blood vessels leading to solid tumor vessel permeability and increased drug delivery [184,243,244,245]. It should be recalled however that several strategies that target tumors inadvertently affect normal tissues as well.

Another effective way to improve drug penetration and efficacy is to inhibit sequestrations of drugs in cellular compartments such as endosomes [246,247,248,249,250,251]. Yet another strategy involve modifying the ECM to enable enhanced penetration of drugs into solid tumors [187,252,253,254]. Caution must be exercised however as ECM modification can promote cancer metastasis [255,256,257,258]. Modifying or degrading even part of the ECM might create a highway through which cancer cells can migrate to other tissues or organs [257,259,260,261].

The ABC transporters are an important mechanism for drug resistance. As mentioned above, ABC transporters play a role in protecting tissues from toxins but they also play a role in the uptake of drugs and delivery to their target molecules. As a result, targeting ABC transporters could be used in the treatment of cancer in future. Great interest has been shown towards the manufacture of anti-ABC drugs. Inhibitors against P glycoprotein may be considered to be the best treatment of cancer and prevention of MDR. Additionally, Chen et al. showed that activation protein kinase D isoform 2 (PKD2) is an important modulator of MDR and P-glycoprotein expression in paclitaxel-treated breast cancer cell lines [262]. The same study also demonstrated that shRNA knockdown of PDK2 in breast cancer cell lines resulted in significant decrease in resistance to anticancer agent paclitaxel [262]. These results suggest that inhibition of MDR and P-gp through the inactivation of PKD2 might be a potential strategy to overcome chemoresistance. However, due to the specificity of the anti-ABC drugs, each patient’s ABC profile and expression levels will need to be determined before treatment using these drugs. The use of antibodies has been successful in increasing the efficacy of anticancer drugs and reducing growth factors which are overexpressed in breast cancer. The use of trastuzumab against the human epidermal growth factor receptor 2 (HER2), a protein involved in the development of breast cancer, was found to increase the efficacy of chemotherapy in metastatic breast cancer that overexpresses HER2 [263]. Another example is the monoclonal antibody cetuximab, which specifically blocks EGFR that is overexpressed in several cancers. Cetuximab is effective in patients who were resistant to treatment with fluorouracil and irinotecan in colorectal cancer [264].

Epithelial-to-mesenchymal transition (EMT) is one of the factors that contributes to chemoresistance and therefore future drug discovery targeting EMT should be considered [62]. Drugs targeting the TM are better potential strategies to overcome chemoresistance. More especially by targeting the hypoxic regions of tumors to improve drug delivery. Using combinational therapies targeting different stromal cells (such as MSCs, CAFs, ECM) found in the TM can enhance the efficacy of many antitumor agents. This can be achieved by understanding the mechanisms of cell-ECM interactions and using drugs that inhibit components involved in ECM remodeling. How chemotherapy affects the TM stromal components is only now becoming clear. Several strategies targeting stroma-initiated signaling are being explored to combat drug resistance. Very few studies, however, have focused on how stromal components respond to chemotherapy and how this contribute to chemoresistance. Recent studies have shown that stroma cells develop drug resistance in the same way as cancer cells, and that stromal cell-drug resistance is vital for cancer cell drug resistance. Targeting the TM and stroma-initiated signaling might be effective ways of killing cancer cells if done in combination with conventional therapy. The protective effect provided by stromal cells to cancer cells can be blocked through selective inhibition of specific receptors.

Lastly, many in vitro models utilized during drug development do not recapitulate the in vivo tumor environment. Most drug development assays are done on 2D cancer cell monolayer cultures where cancer cells are fully exposed to chemotherapeutic reagents do not show drug resistance. Of late, several 3D models have been developed to study cancer cell behavior in vitro. Multicellular tumor spheroids are being used as in vitro tumors and novel information is being obtained. While 3D culture of cancer cells recapitulate the in vivo tumor environment better than 2D culture, cancer cell drug resistance in 3D culture is not only due to cellular changes. Drug distribution in 3D is affected by many other factors such as the presence of ECM components and soluble factors within the microenvironment milieu. The involvement of biomedical engineers in the development of 3D culture models is important since many of these models take into account ECM biophysical properties and controllability in designing the best model. Finally, to fully recapitulate the 3D setting it will be important to include a vascular component such as endothelial cells.

## 5. Conclusions

The future success of cancer therapy is dependent in part on the ability to identify and target mechanisms and pathways involved in chemotherapy resistance. Several targeted strategies including the use of monoclonal antibodies still require a proper understanding of chemoresistance before successful treatment is achieved. Strategies to inhibit processes such as EMT and the removal of supporting stromal cells and the ECM are some of the ways being envisaged to treat cancer in the future. Importantly, the role of the TM components in tumor development and metastasis is now under greater scrutiny. The efficacy of chemotherapy is impaired by reduced delivery of drugs to tumor cells leading to resistance of many anticancer agents. Drug inactivation, inhibition of apoptosis, EMT and the TM play an essential role in events leading to drug resistance and relapse. Tumor associated ECM also plays a role in chemoresistance by providing an environment that stimulates survival pathways. Unlike tumor cells, the components of the TM do not harbor genetic mutations. TM- or stromal directed therapies appear to be gaining ground as they can be used in combination with conventional therapies to control malignant progression. Unfortunately, as many studies have shown, several therapeutic targets identified in stromal cells are common to tumor cells as well, presenting a huge conundrum to scientists. Clinical trials targeting a dysregulated TM show some avenues that can be taken to engineer stromal cells to modulate conventional therapeutic efficacy. Therefore, multiple drugs targeting the TM and inhibiting tumor-stroma interactions may be important strategies to overcome chemoresistance and improve cancer treatment.
